# ColoLipidGene: signature of lipid metabolism-related genes to predict prognosis in stage-II colon cancer patients

**DOI:** 10.18632/oncotarget.3130

**Published:** 2015-02-06

**Authors:** Teodoro Vargas, Juan Moreno-Rubio, Jesús Herranz, Paloma Cejas, Susana Molina, Margarita González-Vallinas, Marta Mendiola, Emilio Burgos, Cristina Aguayo, Ana B. Custodio, Isidro Machado, David Ramos, Meritxell Gironella, Isabel Espinosa-Salinas, Ricardo Ramos, Roberto Martín-Hernández, Alberto Risueño, Javier De Las Rivas, Guillermo Reglero, Ricardo Yaya, Carlos Fernández-Martos, Jorge Aparicio, Joan Maurel, Jaime Feliu, Ana Ramírez de Molina

**Affiliations:** ^1^ Molecular Oncology, IMDEA-Food Institute, CEI UAM+CSIC, Madrid (Spain); ^2^ Translational Oncology Laboratory, La Paz University Hospital (IdiPAZ), Madrid (Spain); ^3^ Precision Oncology Laboratory (POL), Infanta Sofía University Hospital, Madrid (Spain); ^4^ Biostatistics and Bioinformatics Unit, IMDEA-Food Institute, CEI UAM+CSIC, Madrid (Spain); ^5^ Grupo Español Multidisciplinar en Cáncer Digestivo (GEMCAD); ^6^ Pathology Department, La Paz University Hospital (IdiPAZ), Madrid (Spain); ^7^ Medical Oncology, La Paz University Hospital (IdiPAZ), Madrid (Spain); ^8^ Pathology Department, Oncologic Institute of Valencia (IVO), Valencia (Spain); ^9^ Molecular Pathology, La Fe University Hospital, Valencia (Spain); ^10^ Gastrointestinal and Pancreatic Oncology, Centro de Investigación Biomédica en Red de Enfermedades Hepáticas y Digestivas (CIBEREHD), Institut d’Investigacions Biomèdiques August Pi i Sunyer (IDIBAPS), Hospital Clínic, Barcelona (Spain); ^11^ Genomics Unit, Science Park, Madrid (Spain); ^12^ Bioinformatics and Functional Genomics Research Group, Cancer Research Center (CSIC-USAL), Salamanca (Spain); ^13^ Medical Oncology, Oncologic Institute of Valencia (IVO), Valencia (Spain); ^14^ Medical Oncology, La Fe University Hospital, Valencia (Spain); ^15^ Medical Oncology and Translational Genomics Group and Targeted Therapeutics, Institut d’Investigacions Biomèdiques August Pi i Sunyer (IDIBAPS), Hospital Clínic, Barcelona (Spain)

**Keywords:** colon cancer, lipid metabolism, biomarker, prognosis

## Abstract

Lipid metabolism plays an essential role in carcinogenesis due to the requirements of tumoral cells to sustain increased structural, energetic and biosynthetic precursor demands for cell proliferation. We investigated the association between expression of lipid metabolism-related genes and clinical outcome in intermediate-stage colon cancer patients with the aim of identifying a metabolic profile associated with greater malignancy and increased risk of relapse. Expression profile of 70 lipid metabolism-related genes was determined in 77 patients with stage II colon cancer. Cox regression analyses using c-index methodology was applied to identify a metabolic-related signature associated to prognosis. The metabolic signature was further confirmed in two independent validation sets of 120 patients and additionally, in a group of 264 patients from a public database. The combined analysis of these 4 genes, ABCA1, ACSL1, AGPAT1 and SCD, constitutes a metabolic-signature (ColoLipidGene) able to accurately stratify stage II colon cancer patients with 5-fold higher risk of relapse with strong statistical power in the four independent groups of patients. The identification of a group of 4 genes that predict survival in intermediate-stage colon cancer patients allows delineation of a high-risk group that may benefit from adjuvant therapy, and avoids the toxic and unnecessary chemotherapy in patients classified as low-risk group.

## INTRODUCTION

Colorectal cancer (CRC) is one of the main causes for morbidity and mortality worldwide, and represents the fourth most common cancer in men and the third in women [1, 2].

Prediction of outcome in CRC is based on the TNM staging classification, which constitutes a good predictor of survival in patients with TNM Stage I and IV with survival rates of 90–95% and < 10%, respectively [3]. However, in patients with intermediate TNM Stages II and III, within survival rates of 70–85% and 40–80% respectively [3], the prediction of outcome is often imprecise with relevant consequences in the clinical outcome and quality of life of patients. Consequently, identification of novel biomarkers that improve the prognostic ability in these CRC stages is needed, and constitutes a main objective of scientific research in the area [4–7].

Recently, altered metabolism has been added to the list of core hallmarks of cancer [8]. It is well known that lipid metabolism plays a crucial role in different types of cancer due to the special requirements of cancer cells to sustain the additional energetic and biosynthetic precursor demands for cell proliferation [9, 10]. These changes in lipid metabolism affect essential cellular processes [10, 11], and overexpression of lipogenic enzymes has been reported as a common characteristic of many cancers [11–13]. In fact, key enzymes involved in lipid-metabolic pathways are differentially expressed in normal and tumoral tissues, and some of them have been individually proposed as prognosis markers in cancer [12]. However, the analysis of dysregulated expression of lipid metabolic enzymes as a whole in carcinogenesis remains to be established [14].

Here, for the first time, gene expression levels of a wide-range of lipid metabolism-related genes to identify different metabolic patterns conferring tumor energetic advantage consequently associated with worse clinical outcome, are analyzed in stage II CRC patients.

## RESULTS

### Global analysis of lipid metabolism-related genes in stage II-CRC patients

With the aim of classifying patients within the same clinicopathological stage according to their molecular metabolic characteristics, we carried out a global and simultaneous analysis of lipid metabolism-related genes in a training group of 77 samples of patients with stage II-CRC patients. Median follow-up of these patients was 71.5 months. The 3-year DFS was 72.3% and we identified local and/or distant recurrence in 22 patients (28.57%), of which 13 patients (59.01%) died of CRC. Thirty patients (38.96%) did not receive adjuvant treatment, whereas 47 patients (61.04%) received chemotherapy based on 5FU-LV (Fluoracil-Leucovorin) treatment. List of 70 lipid metabolism-related genes selected by their crucial regulatory role of lipid pathways and their involvement in different aspects of lipid metabolism is shown in Table 1. Detailed clinico-histopathological characteristics of these patients are summarized in Table 2.

**Table 1:** Lipid metabolism-related genes include in the study HR (95% CI): genes with significant association with tumor progression in the training group.

Metabolic Pathway	Symbol	Gene name	Chromosome location	HR (95% CI)	*P*
Adipocytokine signaling and immune homeostasis	ADIPOQ	Adiponectin, C1Q and collagen domain containing	3q27		
	CFI	Complement factor I	4q25		
	PPARGC1A	Peroxisome proliferator-activated receptor gamma, coactivator 1 alpha	4p15.1		
	SLC2A4	Solute carrier family 2 (facilitated glucose transporter), member 4	17p13		
Bile acid biosynthesis	ABCB11	ATP-binding cassette, sub-family B (MDR/TAP), member 11	2q24		
	SLC10A2	Solute carrier family 10 (sodium/bile acid cotransporter family), member 2	13q33		
	SLCO1A2	Solute carrier organic anion transporter family, member 1A2	12p12		
	SLCO1B1	Solute carrier organic anion transporter family, member 1B1	12p		
Endocytosis of specific ligands	LDLR	Low density lipoprotein receptor	19p13.2		
Fatty acid biosynthesis	ACACA	Acetyl-Coenzyme A carboxylase alpha	17q21		
	FADS1	Fatty acid desaturase 1	11q12.2–q13.1		
	FADS2	Fatty acid desaturase 2	11q12.2	0.39 (0.16–0.93)	0.0298
	FADS3	Fatty acid desaturase 3	11q12–q13.1		
	FADS6	Fatty acid desaturase domain family, member 6	17q25.1		
	FASN	Fatty acid synthase	17q25		
	SCD	Stearoyl-CoA desaturase (delta-9-desaturase)	10q24.31	3.57 (1.06–12.08)	0.0181
	SCD5	Stearoyl-CoA desaturase 5	4q21.22		
Fatty acid β-oxidation	ACADM	Acyl-Coenzyme A dehydrogenase, C-4 to C-12 straight chain	1p31	4.39 (1.03–18.8)	0.0152
	ACAT1	Acetyl-Coenzyme A acetyltransferase 1	11q22.3		
	ACLY	ATP citrate lyase	17q21.2		
	ACSM4	Acyl-CoA synthetase medium-chain family member 4	12p13.31		
	ACSS2	Acyl-CoA synthetase short-chain family member 2	20q11.22		
	ECHS1	Enoyl Coenzyme A hydratase, short chain, 1, mitochondrial	10q26.2–q26.3		
	HADH	Hydroxyacyl-Coenzyme A dehydrogenase	4q22–q26		
	HMGCL	3-Hydroxymethyl-3-methylglutaryl-Coenzyme A lyase	1p36.1-p35	2.83 (1.04–7.69)	0.0271
	HMGCS2	3-Hydroxy-3-methylglutaryl-Coenzyme A synthase 2 (mitochondrial)	1p13-p12	3.81 (0.89–16.31)	0.031
	PPA1	Pyrophosphatase (inorganic) 1	10q11.1–q24		
	SLC25A20	Solute carrier family 25 (carnitine/acylcarnitine translocase), member 20	3p21.31		
Lipid metabolism in peroxisomes	ACOT8	Acyl-CoA thioesterase 8	20q13.12		
	ACOX2	Acyl-Coenzyme A oxidase 2, branched chain	3p14.3	2.9 (1.21–6.93)	0.014
	ACOX3	Acyl-Coenzyme A oxidase 3, pristanoyl	4p15.3	2.4 (1.03–5.58)	0.0474
	ACSL1	Acyl-CoA synthetase long-chain family member 1	4q35	2.93 (1.26–6.81)	0.0128
	ACSL3	Acyl-CoA synthetase long-chain family member 3	2q34–q35		
	ACSL4	Acyl-CoA synthetase long-chain family member 4	Xq22.3–q23	4.92 (2.09–11.62)	0.0003
	AGPS	Alkylglycerone phosphate synthase	2q31.2		
	AMACR	Alpha-methylacyl-CoA racemase	5p13		
	FAR1	Fatty acyl CoA reductase 1	11p15.2		
	FAR2	Fatty acyl CoA reductase 2	12p11.22		
	GNPAT	Glyceronephosphate O-acyltransferase	1q42		
	HSD17B4	Hydroxysteroid (17-beta) dehydrogenase 4	5q21	2.64 (1.11–6.29)	0.025
	SCP2	Sterol carrier protein 2	1p32		
Phospholipids metabolism	LIPH	Lipase, member H	3q27		
	MBOAT1	Membrane bound O-acyltransferase domain containing 1	6p22.3		
	MBOAT2	Membrane bound O-acyltransferase domain containing 2	2p25.1		
PPAR signaling	CYP7A1	Cytochrome P450, family 7, subfamily A, polypeptide 1	8q11-q12		
	FABP4	Fatty acid binding protein 4, adipocyte	8q21		
	PLIN1	Perilipin 1	15q26		
	PPARD	Peroxisome proliferator-activated receptor delta	6p21.2		
	PPARG	Peroxisome proliferator-activated receptor gamma	3p25		
Cholesterol transport	ABCA1	ATP-binding cassette, sub-family A (ABC1), member 1	9q31.1	3.08 (1.25–7.56)	0.010
	ABCG5	ATP-binding cassette, sub-family G (WHITE), member 5	2p21		
Triacylglycerol metabolism	AGPAT1	1-Acylglycerol-3-phosphate O-acyltransferase 1 (lysophosphatidic acid acyltransferase, alpha)	6p21.3	4.31 (1.8–10.32)	0.0008
	AGPAT2	1-Acylglycerol-3-phosphate O-acyltransferase 2 (lysophosphatidic acid acyltransferase, beta)	9q34.3	3.37 (1.45–7.81)	0.0052
	AGPAT3	1-Acylglycerol-3-phosphate O-acyltransferase 3	21q22.3	3.78 (1.28–11.17)	0.0068
	AGPAT4	1-Acylglycerol-3-phosphate O-acyltransferase 4 (lysophosphatidic acid acyltransferase, delta)	6q26		
	AGPAT5	1-Acylglycerol-3-phosphate O-acyltransferase 5 (lysophosphatidic acid acyltransferase, epsilon)	8p23.1		
	DGAT1	Diacylglycerol O-acyltransferase homolog 1 (mouse)	8q24.3		
	LIPG	Lipase, endothelial	18q21.1		
	MGLL	Monoglyceride lipase	3q21.3		
Regulation of the hepatocyte growth factor (HGF)	SPINT1	Serine peptidase inhibitor, Kunitz type 1	15q15.1	3.96 (1.17–13.41)	0.010
	ST14	Suppression of tumorigenicity 14 (colon carcinoma)	11q24-q25		
Receptors and basement membrane glycoproteins	MC3R	Melanocortin 3 receptor	20q13.2-q13.3		
	MC4R	Melanocortin 4 receptor	18q22		
	NID1	Nidogen 1	1q43	2.76 (0.93–8.17)	0.043
	NID2	Nidogen 2 (osteonidogen)	14q22.1		
Biosynthesis of metabolic components	GCG	Glucagon	2q36-q37		
	MGAT1	Mannosyl (alpha-1,3-)-glycoprotein beta-1,2-N-acetylglucosaminyltransferase	5q35		
	NAMPT	Nicotinamide phosphoribosyltransferase	7q22.3		
Fatty acid and guanine nucleotide-binding proteins	FABP2	Fatty acid binding protein 2, intestinal	4q28-q31		
	GNB3	Guanine nucleotide binding protein (G protein), beta polypeptide 3	12p13		

**Table 2:** Detailed clinical and histopathological characteristics of patients included in the study

Characteristics	Stage II CRC								
	Training group			Validation group I			Validation group II		
	n° of Patients (%)			n° of Patients (%)			n° of Patients (%)		
**Total sample size (n)**		77	(100)		119	(100)		120	(100)
**Age at Diagnosis (years)**									
Mean	68·22			66·08			—		
Median	69			66			—		
Age Range	32–86			26–91			33–88		
≤50		3	(3·90)		15	(12·60)		15	(12·5)
50–70		39	(50·65)		58	(48·74)		63	(52·5)
≥70		35	(45·45)		46	(38·66)		42	(35)
**Sex**									
Female		33	(42·86)		54	(45·38)		60	(50)
Male		44	(57·14)		65	(54·62)		60	(50)
**Stage**									
IIA (T3 N0 M0)		56	(72·73)		70	(58·82)		99	(82·5)
IIB (T4 N0 M0)		21	(27·27)		49	(41·18)		21	(17·5)
**Regional Lymph Node Metastasis**									
No Lymph node involvement (N0)		77	(100)		119	(100)		120	(100)
1–3 Lymph node involvement (N1)									
≥4 Lymph node involvement (N2)									
Could not be assessed (Nx)									
**Total Lymph Nodes Resected**									
Mean Lymph nodes resected	12·09			14·20		17·38			
Range of Lymph nodes examined	1–29			0–43		3–43			
≤12		46	(59·7)		54	(45·4)		32	(26·67)
>12		30	−39		62	(52·1)		87	(72·5)
Unknown		1	(1·3)		3	(2·5)		1	(0·83)
**Location of Primary**									
Cecum and Ileocecal Valve		2	(2·6)		13	(10·92)		9	(7·5)
Acending colon and Hepatic flexure		29	(37·66)		29	(24·37)		24	(20)
Transverse colon		6	(7·79)		6	(5·04)		6	(5)
Splenic flexure and Descending colon		5	(6·49)		17	(14·29)		6	(5)
Sigmoid colon and rectosigmoid junction		34	(44·16)		54	(45·38)		75	(62·5)
Rectum		1	(1·3)		0			0	
**Grade/Differentiation**									
Well		5	(6·49)		10	(8·4)		11	(9·17)
Moderately		66	(85·71)		95	(79·8)		101	(84·16)
Poor		5	(6·49)		10	(8·4)		8	(6·67)
Unknown		1	(1·3)		4	(3·4)		0	
**Bowel Obstruction/Perforation**									
Yes		10	(12·99)		45	(37·82)		30	(25)
No		67	(87·01)		74	(62·18)		90	(75)
**Other Histological Features**									
Perineural invasion		12	(15·58)		25	(21)		20	(16·67)
Vascular invasion		22	(28·57)		31	(26·05)		14	(11·67)
**Adjuvant treatment**									
5FU-LV^*^		47	(61·04)		0			0	
Xelox/Folox		0			76	(63·87)		41	(34·17)
No treatment		30	(38·96)		43	(36·13)		79	(65·83)
**Disease-free survival**									
Patients with recurrence		22	(28·57)		18	(15·13)		21	(17·5)
**Overall survival**									
nº of Exitus		13	(16·88)		11	(9·24)		15	(12·5)

* 5-Fluorouracil (5FU)-Leucovorin (LV)

Results showed that 16 out of the 70 genes analyzed in this study were differentially distributed in the tumors with a putative association between expression levels and worse clinical outcome in this training group of stage II CRC patients (Table 1), suggesting that specific pathways of lipid metabolism might be specially related to increased malignancy.

### Development of a metabolic-related gene expression signature

**Figure 1:**
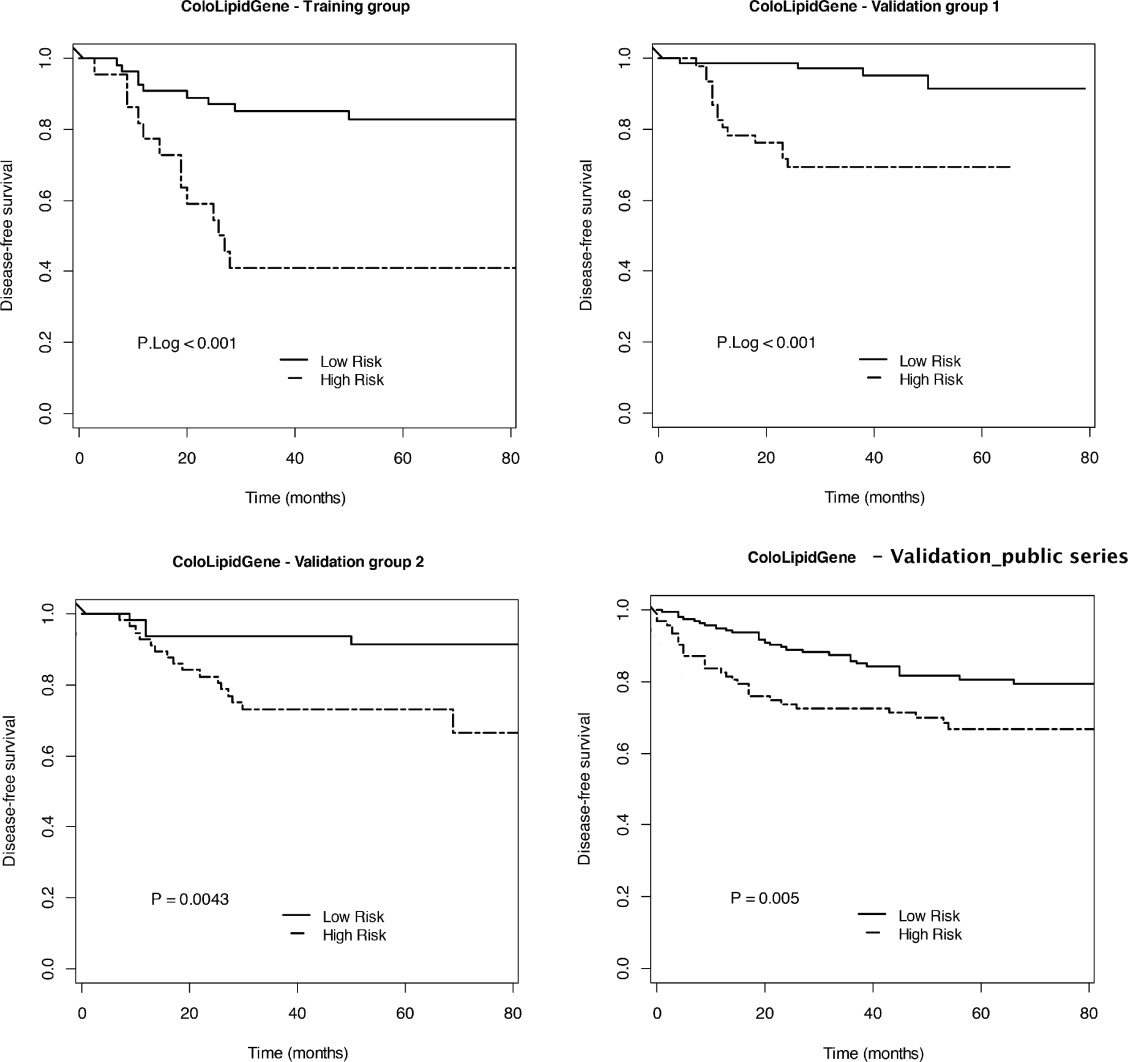
4-gene expression signature to predict DFS in early-stage CRC patients Kaplan-Meier plots for 4-gene expression signature ColoLipidGene and p Log Rank value in the training group, validation groups I and II, and GSE39582 series from Gene Expression Omnibus Database are shown. (Training group: Low risk *n* = 55, High risk *n* = 22; Validation group I: Low risk *n* = 72, High risk *n* = 47; Validation group II: Low risk *n* = 63, High risk *n* = 57; Validation in public GSE39582 series: Low risk *n* = 165, High risk *n* = 95).

The putative interactions among the different genes and the combination of different metabolic patterns were assayed, and models constituting a prognostic signature were constructed selecting the multivariate model with largest c-index (0.72) using 100 times 5-fold cross-validation (CV). A gene expression signature composed of the combination of 4 lipid metabolism-related genes was selected due to its high score to predict DFS, and designated as ColoLipidGene signature. ColoLipidGene, constituted by the combination of ABCA1, ACSL1, AGPAT1 and SCD, genes involved in lipid transport, fatty acid activation and phospholipid-related signaling, was able to significantly predict risk of relapse of CRC patients within the same stage II with a HR (95% CI): 4.65 (1.98–10.93), log-rank *p* < 0.001 (Figure 1). 3-year DFS in patients from High risk group classified by ColoLipidGene was 41% (95% CI: 0.25–0.68) compared with 85% (95% CI: 0.76–0.95) in patients from low risk group. To evaluate whether ColoLipidGene might constitute an independent prognostic classifier, clinical and histopathological data were included in both univariate and multivariate Cox regression analyses. In univariate analysis, tumor size (T), vascular invasion, perineural invasion and bowel obstruction/perforation were the strongest clinical variables associated with prognosis in these patients (Table 3), which were included in the multivariate analysis, together with age > 70 as main nonmodifiable risk factor. Results obtained in the multivariate analysis revealed that ColoLipidGene was an independent prognostic classifier for DFS with 4-fold increased risk of relapse for stage II CRC patients positive for this molecular test [HR (95% CI): 3.94 (1.54–10.11), *p* = 0.005; Table 4], establishing an association between high expression levels of these four genes that constituted ColoLipidGene and worse clinical outcome in these stage II CRC patients.

**Table 3:** Univariate cox regression analysis for disease-free survival of the clinical parameters in stage II CRC patients

Variable		Univariate analysis					
		Training group		Validation group I		Validation group II	
		HR (95% CI)	*P*	HR (95% CI)	*P*	HR (95% CI)	*P*
Age (continuous)		1 (0·96–1·05)	0·839	1·01 (0·98–1·05)	0·492	1 (0·96–1·04)	0·97
Age, > v ≤ 70		1·08 (0·46–2·52)	0·863	1·37 (0·53–3·54)	0·525	0·88 (0·34–2·26)	0·786
Sex, male v female		1·86 (0·76–4·56)	0·163	1·48 (0·57–3·82)	0·413	0·37 (0·14–0·96)	0·032
**Location of primary**			0·321		0·845		0·123
(Reference: Sigmoid colon and rectosigmoid junction)	Cecum and Ileocecal Valve	NA		0·81 (0·18–3·7)		0·48 (0·06–3·6)	
	Acending colon and hepatic flexure	0·77 (0·28–2·07)		0·57 (0·16–2·07)		0·81 (0·27–2·43)	
	Transverse, splenic flexure and descending colon	1·57 (0·54–4·53)		0·79 (0·22–2·86)		NA	
	Rectum						
**Grade/Differentiation**			0·585		0·457		0·319
(Reference: Moderate)	Poor	2·07 (0·48–8·92)		1·51 (0·34–6·68)		2·36 (0·69–8·05)	
	Well	0·63 (0·08–4·72)		2·31 (0·66–8·1)		0·5 (0·07–3·74)	
pT (T4 v T3)		1·75 (0·73–4·17)	0·223	3·19 (1·2–8·49)	0·016	3·42 (1·42–8·27)	0·011
Mean lymph nodes resected (continuous)		1·01 (0·95–1·08)	0·759	0·97 (0·91–1·04)	0·435	0·92 (0·85–0·99)	0·012
Range of lymph nodes examined, > v ≤ 12		1·74 (0·76–4·02)	0·196	0·56 (0·22–1·46)	0·230	0·27 (0·11–0·63)	0·003
Adjuvant treatment, yes v no		1·03 (0·43–2·45)	0·952	1·27 (0·45–3·58)	0·642	1·83 (0·54–6·26)	0.173
Vascular invasion, yes v no		1·08 (0·44–2·65)	0·866	3·08 (1·22–7·78)	0·020	4·44 (1·78–11·04)	0·004
Perineural invasion, yes v no		1·16 (0·39–3·43)	0·793	3·04 (1·2–7·72)	0·025	4·14 (1·69–10·13)	0·004
Bowel obstruction/perforation, yes v no		3·07 (1·19–7·87)	0·034	1·59 (0·63–4)	0·328	4·15 (1·76–9·79)	0·002
Clinical classifier (ASCO risk^*^), High v Low risk		2·53 (0·59–10·81)	0·157	4·35 (0·6–554·13)	0·187	5·52 (1·28–23·73)	0·004

Abbreviations: NA, not available; HR (95% CI), hazard ratio and corresponding 95% confidence interval from univariate cox proportional hazards analysis; P, *p* value from univariate cox regression analysis; ASCO, American Society of Clinical Oncology.

* Patients are considered high risk if they have any of the following events: numbers of lymph nodes examined ≤ 12; T4; poor histologic grade; emergency presentation with obstruction or perforation; perineural or lymphovascular invassion.

**Table 4:** Uni- and multivariate cox regression analysis for disease-free survival of ColoLipidGene signature and individual composing genes, and the clinical classifier (ASCO clinical risk criteria) in stage II CRC patients

Variable	Training group								Validation group I								Validation group II							
	Low Risk		High Risk		Unadjusted		Adjusted#		Low Risk		High Risk		Unadjusted		Adjusted#		Low Risk		High Risk		Unadjusted		Adjusted#	
	*R*	*N*	*R*	*N*	HR(95% CI)	*P*	HR(95% CI)	*P*	*R*	*N*	*R*	*N*	HR(95% CI)	*P*	HR(95% CI)	*P*	*R*	*N*	*R*	*N*	HR(95% CI)	*P*	HR(95% CI)	*P*
ABCA1	7	43	15	34	3·08 (1·25–7·56)	0·010	3·76 (1·4–10·08)	0·006	6	65	12	54	2.63 (0.99–7)	0.045	3.12 (1.1–8.82)	0.026	3	47	18	73	4.21 (1.24–14.32)	0.007	5.05 (1.44–17.65)	0.003
ACSL1	10	51	12	26	2.93 (1.26–6.81)	0.013	2.34 (0.91–6.02)	0.082	5	61	13	58	3.12 (1.11–8.76)	0.021	3.86 (1.16–12.79)	0.017	2	29	19	91	3.35 (0.78–14.4)	0.056	4.89 (1.04–22.97)	0.018
AGPAT1	8	51	14	26	4.31 (1.8–10.32)	0.001	3.54 (1.39–9)	0.007	4	52	14	67	3.11 (1.02–9.47)	0.03	4.45 (1.31–15.11)	0.009	3	40	18	80	3.24 (0.95–11)	0.032	3.11 (0.89–10.93)	0.049
SCD	3	26	19	51	3·57 (1·06–12·08)	0·018	3·13 (0·9–10·93)	0·046	3	57	15	62	5.32 (1.54–18.38)	0.002	4.08 (1.12–14.9)	0.017	12	83	9	37	1.76 (0.74–4.18)	0.208	2.17 (0.85–5.57)	0.113
Clinical classifier (ASCO risk^*^); High v Low risk	2	14	20	63	2·53 (0·59–10·81)	0·157			0	13	18	106	4·35 (0·6–554·13)	0·187			2	40	19	80	5.52 (1.28–23.73)	0.004		
ColoLipidGene; High v Low risk	9	55	13	22	4.65 (1.98–10.93)	< 0·001	3.94 (1.54–10.11)	0·005	4	72	14	47	6.57 (2.15–20.02)	< 0·001	6.55 (2.06–20.75)	< 0·001	5	63	16	57	3.88 (1.42–10.59)	< 0·005	6.89 (2.05–23.19)	< 0·001

Abbreviations: HR (95% CI), hazard ratio and corresponding 95% confidence interval from adjusted or unadjusted Cox regression analyses; P, p value from adjusted or unadjusted Cox regression analyses; N, Nº of patients in each risk group; R, Nº of patients with relapse; ASCO, American Society of Clinical Oncology.

* Patients are considered high risk if they have any of the following events: numbers of lymph nodes examined ≤ 12; T4; poor histologic grade; emergency presentation with obstruction or perforation; perineural or lymphovascular invassion.

# Cox regression analyses were adjusted for T stage, Vascular invassion, Perineural invassion, Bowel Obstruction/Perforation and Age > 70.

### ColoLipidGene validation

In order to confirm the association of these four lipid metabolism-related genes with the potential aggressiveness of the tumor, we evaluated lipidic gene expression analysis as previously indicated in an independent validation set of 119 stage II CRC patients (validation group I). Median follow-up and 3-year DFS of these patients was 43 months and 86.1% respectively. Eighteen out of the 119 patients relapsed with local and/or distant metastasis (15.3%), of which 11 patients (61.1%) died due to CRC. Forty-three patients (36.13%) did not receive adjuvant treatment, whereas 76 patients (63.87%) received chemotherapy based on Xelox or Folfox4 treatment Table 2). Gene expression analysis in the validation group confirmed the potential value of the 4-gene expression signature ColoLipidGene (c-index = 0.77) as a prognostic biomarker to identify stage II CRC patients at high risk of relapse [HR (95% CI): 6.57 (2.15–20.02), log-rank *p* < 0.001; Figure 1, Table 4]. Thus, 3-year DFS in patients from High risk group classified by this gene expression profile was 69% (95% CI: 0.57–0.84) compared with 97% (95% CI: 0.93–1) in patients from low risk group in the validation group 1. The multivariate analysis also confirmed ColoLipidGene as an independent prognostic classifier for DFS in the validation group I with 6.5-fold increased risk of relapse for stage II CRC patients [HR (95% CI): 6.55 (2.06–20.75), *p* < 0.001; Table 4].

In order to further validate the prognostic strengthen of CololipidGene, we analyzed the expression levels of the metabolic-related gene signature in an independent set of 120 stage II CRC patients (validation group II) from hospitals located in different regions (Clinic University Hospital in Barcelona, La Fe University Hospital and Oncologic Institute of Valencia). Median follow-up and 3-year DFS of this group of 120 patients was 58.3 months and 84% respectively. Twenty-one out of the 120 patients relapsed with local and/or distant metastasis (17.5%), of which 15 patients (71.43%) died due to CRC. 79 patients (65.83%) did not receive adjuvant treatment, whereas 41 patients (34.17%) received chemotherapy (Table 2). As it is shown in Figure 1, ColoLipidGene prognostic value was further confirmed in this group of patients (c-index = 0.7), identifying stage II CRC patients with almost 7-fold higher risk of relapse 6.89 [HR (95% CI): 6.89 (2.05–23.19), *p* < 0.001; Table 4]. Similarly to the previously examined validation group 1, the 3-year DFS rates in the validation group 2 in patients from High risk group classified by ColoLipidGene was 73% (95% CI: 0.63–0.86) compared with 94% (95% CI: 0.88–1) in patients from low risk group in this group of stage II CRC patients.

**Figure 2:**
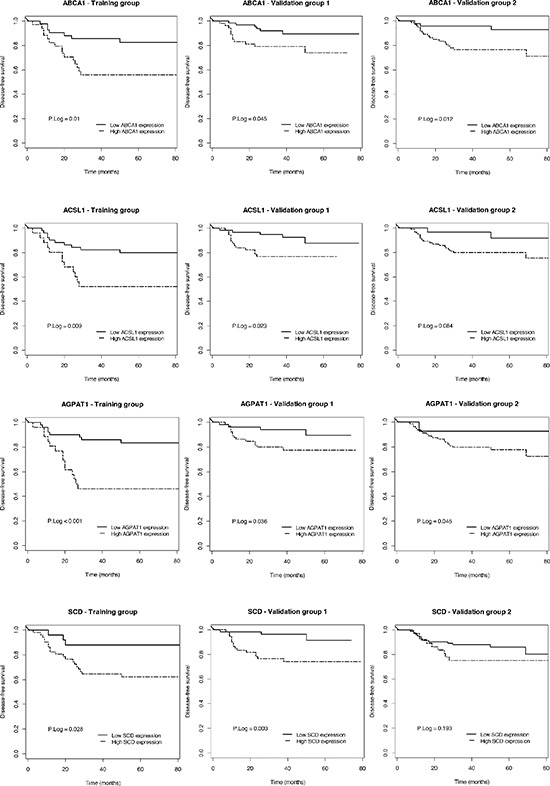
Prognostic value of individual genes constituting ColoLipidGene in the different group of patients Kaplan-Meier plots for individual genes of ColoLipidGene and p Log Rank value in the training group and validation groups I and II are shown.

Thus, ColoLipidGene was revealed as an independent prognostic classifier for DFS in all groups of stage II CRC patients, showing stronger power and accuracy than any other variables, including the currently used clinical classification. Of note, though to a lower extent, all genes defining ColoLipidGene combined biomarker constitutes individual biomarkers of prognosis of stage II-CRC patients (Figure 2), facilitating the interpretation of the results. Thus, after adjusting for potential confounding factors, the increased risk of relapse (pooled hazard ratios based on random-effects meta-analysis) for patients with increased levels of ABCA1, ACSL1, AGPAT1 or SCD was of HR (95% CI): 3.78 (2.03–7.03), *p* < 0.001; HR (95% CI): 3.14 (1.61–6.13), *p* < 0.001; HR (95% CI): 3.65 (1.93–6.91), *p* < 0.001; and HR (95% CI): 2.81 (1.46–5.38), *p* = 0.002 respectively. This is, higher levels of either ABCA1, ACSL1, AGPAT1 or SCD is associated with worse clinical outcome of the patients as independent molecular factors, further supporting the strength of their combined analysis as ColoLipidGene metabolic biomarker.

Additionally, to further validate the prognosis prediction ability of ColoLipidGene, we evaluate its prognostic value in public gene expression data of colon cancer patients from Gene Expression Omnibus Database. The public series was constituted for 566 patients with colon cancer, 264 out of the 566 with stage II colon cancer, including local and/or distant recurrence in 59 patients (22.34%). As it is shown in Figure 1, ColoLipidGene prognostic power was further confirmed in this group of independent stage II colon cancer patients (p Log Rank value < 0.005), identifying patients with almost 2-fold higher risk of relapse [HR (95% CI): 2.05 (1.23–3.42), *p* = 0.006]. 3-year DFS in patients from High risk group classified by ColoLipidGene was 73% (95% CI: 0.64–0.82) compared with 86% (95% CI: 0.80–0.92) in patients from Low risk group in these group of stage II colon cancer patients from public database. These results obtained in four independent groups of stage II colon cancer patients obtained in different time points and locations, confirm the potential use of these metabolic biomarker as a prognostic tool in early-stage colon cancer patients.

## DISCUSSION

Currently, outcome prediction in CRC is based on TNM or Dukes staging classification system, insufficient for accurately predicting survival of stage II-CRC patients, in which chemotherapy administration is one of the decision to make in patient management. Thus, new biomarkers to better stratify and correctly prescribe the best treatment for precise patients to optimize outcome, reduce adverse toxicity events, reduce cost-effectiveness ratios is a necessary demand for early-stage CRC patients.

Several studies have recently proposed complex gene expression profiles to predict OS and DFS in CRC patients, but neither of them is focused on lipid metabolism [4–7]. Most of these proposed molecular biomarkers contain a large number of genes that might complicate the experimental application, with limited biological significance of the combination of genes that might also complicate the interpretation of results. Wang and colleagues developed a 23-gene expression signature studied in 74 stage II-CRC [15]. However, a completely different 30-gene expression signature was proposed by Barrier and colleagues in another group of stage II colon cancer patients by using the same Genechip and different results were obtained when they studied the predictive value of the 23-gene expression signature proposed by Wang and colleagues in their patients [16]. Oncotype Dx Colon Cancer test is based on mRNA expression of 12 target genes, and was validated in a large clinical trial of primary CRC cases, finding clinical utility when used as a complement to T stage and mismatch repair status, specifically for patients who have T3, MMR-proficient, stage II disease [17, 18]. The ColoPrint gene signature uses 18 genes on a microarray platform. The classifier was applied in a training set (*n* = 188) of stage I, II and III, but they didn’t show significant prognostic value for DFS within each stage, and the robustness of the signature is awaiting evaluation in a prospective trial [6]. Currently, only one prognosis test for stage II and III CRC patients, the ColoGuidePro, has been designed with less than 8 genes. However, this 7-gene expression signature only stratified stage III CRC patients, whereas prediction of relapse of stage-II patients was not validated [7]. Thus, evidence stand out the necessity of understanding the biological significance of a biomarker to properly applied obtained information, and recent research has been focused on defining molecular subtypes (including “mesenchymal” and “stem cell”) [19]. In this sense, different metabolic profiles might provide not only information regarding molecular subtypes related to energetic capacity of tumoral cells, but also might provide new therapeutic options involving the inhibition of these pathways, pointing at the putative clinical relevance of this study.

Altered cellular metabolism is considered an important hallmark of cancer [8] and several enzymes involved in lipid metabolism have been shown to be involved in tumor malignancy [11, 12]. Here, we analyzed for the first time the putative association between global expression of lipid metabolism-related genes and prediction of outcome in early-stage CRC patients. Results from three different groups of patients recruited within different time points and by hospitals located in different regions, and from public whole human genome Microarray data of colon cancer patients from Gene Expression Omnibus Database, point at activation of ABCA1, ACSL1, AGPAT1 and SCD as main relevant metabolic factors in malignant progression. ATP-Binding Cassette Subfamily-A Member 1 (ABCA1), identified as a major regulator of phospholipid homeostasis, is involved in transport of cellular cholesterol from peripheral cells and tissues. The expression of ABC transporters (including ABCA1) have been associated with deregulation in one of the most drug-resistant cancers, the pancreatic ductal adenocarcinoma (PDAC) [20]. In addition, reported data suggest that ABCA1 gene might contribute to a more aggressive growth of multiple drug resistant melanomas [21], and the individual association of ABCA1 with a more aggressive phenotype of colorectal tumors has been also identified in an additional study focused on the relationship between metabolic syndrome and colorectal cancer (Vargas T et al., 2014) [22]. On the other hand, ACSL1 is an isozyme of Acyl-CoA synthetase (ACS) family, known to play an important role in lipid metabolism, cancer cell survival and apoptosis inhibition [11]. ACS converts long-chain fatty acids to acyl-CoA, a crucial step in several lipid metabolism pathways. Previous reports have indicated that other ACS isozymes, such as ACSL4 and ACSL5, are overexpressed in various types of cancer, including colon adenocarcinoma [11, 14, 23–27]. Namely, Triacsin C (a potent inhibitor of ACS, including ACSL1 [28]) induce massive apoptosis and selective cytotoxicity in cancer cells [11]. AGPAT1 encodes an enzyme that converts lysophosphatidic acid (LPA) into phosphatidic acid (PA), phospholipids involved in signal transduction and in lipid biosynthesis. While several studies have suggested the association between enhanced transcription of AGPAT2 and certain cancers or inflammation-associated diseases, neither of them have described the influence of AGPAT1 isoform on cancer prognosis [29]. Finally, the products of SCD (Stearoyl-CoA-desaturase 1) represent important precursors for the formation of complex lipids, and human SCD was found to be up-regulated in transformed cells and overexpressed in a variety of human cancers, being recently proposed as a potential target for cancer therapy [30]. These results suggest that activation of lipid metabolism through different metabolic steps is an essential event to facilitate early-stage tumor progression, probably due to both structural and energetic requirements of tumoral cells, as it has been previously proposed [12]. Thus, the combined activation of these four genes might ensure tumoral cells a competitive advantage through a quick supply of metabolic-related precursors through fatty acid and cholesterol metabolisms, and on the other hand avoiding lipidic-related toxicity through the alteration of the desaturase SCD.

We have identified a gene expression signature constituted of only 4 genes, as an independent prognostic biomarker of tumor progression for stage-II CRC patients, that exhibited better prognostic prediction within the same pathological stage even when compared with American Society of Clinical Oncology (ASCO) clinical risk criteria, suggesting its potential relevance as a complementary approach in clinical decision-making for this group of patients. Though further *in vitro* and *in vivo* work has still to be done to understand the contribution of ABCA1, ACSL1, AGPAT1 and SCD in tumor progression, it is important to note that the prognostic prediction ability of this profile was confirmed in different sets of cancer patients, including an independent series of public gene expression microarray data of colon cancer patients from Gene Expression Omnibus Database, reinforcing the relevance of ColoLipidGene as a reliable prognostic tool in stage II colon cancer patients. These results together with the reduced number of genes constituting the signature, the advantage of ColoLipidGene vs the other identified signatures lies on the fact that the four genes constituting ColoLipidGene are involved in a specific biological process (lipid metabolism), establishing an hypothesis that support the role of this signature in the aggressiveness of the tumors. Finally, ColoLipidGene is constituted by key metabolic “druggable” enzymes, pointing at them as main promising therapeutic targets for these patients.

## MATERIALS AND METHODS

### Study design and patients

80 stage II CRC patients undergoing surgery between 2000 and 2004 in La Paz University Hospital were enrolled in the training group for this retrospective study. Three of them were discarded because of the quality material obtained. Inclusion criteria: Age ≥ 18, completely resected rectal cancer or colon adenocarcinoma located at ≥ 15 cm of the anal verge as determined by endoscopy or above the peritoneal reflection in the surgical resection, confirmed Stage II AJCC/UICC primary CRC and follow-up of at least 36 months. Exclusion criteria: death within 30 days after surgery, other cancers in previous 5 years and inflammatory bowel disease or specific gene-related cancer.

We validated the results in two different sets of patients. The validation group I was composed of 119 stage II CRC patients recruited in different time period (between 2004 and 2008) from La Paz University Hospital (Madrid). Results were externally validated in an additional set of 120 patients with stage II CRC (validation group II) from Clinic University Hospital (Barcelona), La Fe University Hospital (Valencia) and the Oncologic Institute of Valencia (IVO). For all groups of patients, Formalin-Fixed, Paraffin-Embedded (FFPE) samples were obtained with the approval of the human research Ethics review Committee of the hospitals involved (HULP-PI-1452). Clinico-histopathological data of patients were prospectively collected on clinical history and were confirmed by oncologists of the hospitals implicated in this study. All FFPE samples were revised by an anatomic pathologist ensuring > 85% of tumoral cells in each sample. Patients in all groups were classified following the clinical risk criteria of American Society of Clinical Oncology (ASCO). Since all patients included were within the same clinicopathological stage II, this clinical classifier was considered as the standard for determining patient prognosis. No other reference standard is applicable in this study since on similar molecular biomarker is currently applied for CRC patients with these characteristics. The reference clinical classifier consider patients with high risk of relapse if any of the following events: numbers of lymph nodes examined ≤ 12; T4; poor histological grade; emergency presentation with obstruction or perforation; perineural or lymphovascular invasion [31].

Additionally, we tested the prognostic power of ColoLipidGene in public whole human genome Microarray data of colon cancer patients from Gene Expression Omnibus Database. Raw gene expression data corresponding to the GSE39582 series was downloaded from the source as original CEL files. The 566 Affymetrix U133Plus2 arrays included in the dataset were processed together locally by using the aroma.affymetrix R package [32]. Robust Multichip Average (RMA) method was applied for background correction and normalization. Thus, we selected the stage II colon cancer patients (*n* = 264) and evaluated the prediction ability of ColoLipidGene in these public gene expression series of colon cancer patients.

### Gene expression assays

Samples were deparaffinated and total RNA was extracted using RNeasy FFPE Kit (Qiagen Gmbh, Germany). 1 μg of total RNA was reverse transcribed by High Capacity cDNA Archive Kit (Applied Biosystems) for 2 h at 37°C. A Taq-Man Low Density Array (Applied Biosystems) was specifically designed for this experiment and was composed of 70 lipid metabolism-related genes (Table 1). These 70 genes within all genes present in pathways related with lipid metabolism were selected due to their key role as master regulators of cell metabolism, key steps of interconnection among lipid pathways or their reported role in biological processes associated with cancer. Gene-expression assays were performed in a HT–7900 Fast Real time PCR. The geometric mean of the internal control genes (GAPDH and B2M) was used as endogenous control. RT-StatMiner software (Integromics® Inc., Madison, USA) was used to detect and determine the quality control and differential expression analyses of data.

### Statistical analysis

The primary endpoint of the study was the relationship between gene expression and disease-free survival (DFS). Quantification of gene expression was calculated with the 2^−ΔCt^ method. Time to relapse was obtained for the analysis of 3-year DFS, defined from the time of surgical procedure. The Kaplan-Meier method was used to estimate DFS. Log-rank test for Univariate Cox regression analysis was performed to test association between DFS and individual gene expression. To assess the effect on survival with adjustment for potential confounding factors, proportional hazards Cox regression modeling was used. Multivariate analysis included only variables that were significant (*p* < 0.05) in the Univariate analysis and age > 70 as main nonmodifiable risk factor. Hazard ratios (HR) and 95% CI were calculated from the Cox regression model. The threshold for dichotomization of the expression data of each gene into a low and a high value was selected based on the cutoff point with largest prediction ability, evaluated by the c-index using 100 times 5-fold cross-validation (CV). Data were blind and independently analyzed by two different groups of experts in the field (Biostatistics and Bioinformatics Unit, IMDEA Food Institute and Bioinformatics and Functional Genomics Research Group, Cancer Research Center (CSIC-USAL).

The prognostic gene expression signature was developed analyzing the prediction ability for all possible Cox regression models with genes found within the range of significance in the univariate analysis, and selecting the multivariate model with largest c-index (> 0.75) implemented using 100 times 5-fold CV [33]. The threshold value was chosen such that the frequency of the high risk group was as the “observed recurrence risk”. Determined threshold value in the training set of patients was further applied to validation sets of patients. All reported *p* values were two-sided. Statistical significance was defined as *p* < 0.05. The statistical analyses were done by use of the R statistical software v2.15 (http://www.r-project.org).
